# The Impact of Complex Oral Rehabilitation on TMJ and Postural Alterations in Patients with Scapulohumeral Fractures

**DOI:** 10.3390/jcm15103597

**Published:** 2026-05-08

**Authors:** Ovidiu Stamatin, Ana Maria Carina Balcos, Tudor Hamburda, Maria Antonela Beldiman, Vlad Stefan Proca, Violina Budu, Liana Aminov, Laura Elisabeta Checherita, Bogdan Petru Bulancea, Eşanu Irina Mihaela, Norin Forna, Ana Elena Sîrghe

**Affiliations:** 1 Department of Oral-Implantology, Dental Prostheses Technology and Removable Dentures, Faculty of Dental Medicine, Grigore T. Popa University of Medicine and Pharmacy, Str. Universității nr. 16, 700115 Iasi, Romania; ovidiu.stamatin@umfiasi.com (O.S.); bulanceabogdan@umfiasi.ro (B.P.B.); 2 Surgery Department, Preventive Medicine, Faculty of Dental Medicine, Grigore T. Popa University of Medicine and Pharmacy, Str. Universității nr. 16, 700115 Iasi, Romania; carina.balcos@umfiasi.com; 3 Department of Odontology, Periodontology and Fixed Prosthesis, Faculty of Dental Medicine, Grigore T. Popa University of Medicine and Pharmacy, Str. Universității nr. 16, 700115 Iasi, Romaniaviolinabudu@umfiasi.ro (V.B.); lianaaminov@yahoo.com (L.A.); 4 Discipline of Medical Semiology, Medical Department I, Grigore T. Popa University of Medicine and Pharmacy of Iași, Str. Universității nr. 16, 700115 Iasi, Romania; irina.esanu@umfiasi.ro; 5 Surgery Department, Discipline of Orthopedics in Faculty of General Medicine, Grigore T. Popa University of Medicine and Pharmacy, Str. Universității nr. 16, 700115 Iasi, Romania; 6 1st Dental Medicine Department, Faculty of Dental Medicine, Grigore T. Popa University of Medicine and Pharmacy, Str. Universității nr. 16, 700115 Iasi, Romania; ana.sirghie@umfiasi.ro

**Keywords:** temporomandibular disorders, scapulohumeral fractures, postural imbalance, TMJ dysfunction, multimodal rehabilitation, occlusal splints, baropodometric analysis, musculoskeletal biomechanics

## Abstract

**Background and Objectives:** Temporomandibular disorders (TMDs) are prevalent conditions affecting the temporomandibular joint and associated musculature, arising from a complex interplay of biomechanical, neuromuscular, and psychosocial factors. Increasing evidence supports functional interconnections among the TMJ, cervical spine, and shoulder girdle, suggesting that dysfunction in one region may influence others; however, these relationships remain incompletely understood. This study aimed to evaluate the association between scapulohumeral trauma, postural abnormalities, and TMDs, and to assess their evolution following interdisciplinary rehabilitation. **Materials and Methods:** A retrospective observational study with prospective follow-up was conducted in patients with scapulohumeral fractures associated with TMD and postural abnormalities. Postural parameters and the clinical features of temporomandibular disorders (TMDs) were evaluated at baseline and follow-up using a structured clinical assessment informed by the DC/TMD framework, together with clinical examination, electromyographic analysis, and mandibular mobility measurements. Postural evaluation was performed using digital baropodometric analysis (Free Med™ platform with FreeStep™ software, standard Medica sensor, Rome, Italy. Patients received individualized multidisciplinary treatment, including orthopaedic rehabilitation, occlusal splint therapy, physiotherapy (twice weekly), pharmacological management, and odonto-periodontal care. Statistical analyses were performed using non-parametric tests (*p* < 0.05). **Results:** Significant postural improvement was observed (*p* < 0.01), with the proportion of patients with normal posture increasing from 0% to 22.2% and the proportion with moderate forward lean decreasing from 53.3% to 15.6%. TMD severity decreased progressively across evaluations (Friedman χ^2^ = 72.35, *p* < 0.01). No statistically significant differences were found between treatment groups with respect to postural outcomes, although descriptive differences in TMD improvement were observed at later evaluation points. **Conclusions:** Interdisciplinary rehabilitation was associated with significant improvements in both postural alignment and TMD severity. Scapulohumeral trauma may be associated with alterations in TMJ function and overall posture, while multimodal therapy supports functional recovery. Further randomized controlled studies are needed to confirm these findings.

## 1. Introduction

Temporomandibular disorders (TMDs) comprise a group of conditions affecting the temporomandibular joint (TMJ), masticatory muscles, and associated structures, typically characterized by pain, restricted mandibular mobility, and joint sounds. With an estimated global prevalence of approximately 34% and a higher incidence among women, TMDs represent a significant clinical and public health concern [1].

Current evidence supports a biopsychosocial framework that emphasizes the interaction between biological, neuromuscular, behavioral, and psychosocial factors in the etiology of TMD [2]. Within this context, the cranio-cervico-mandibular system describes the biomechanical and neuromuscular integration linking the TMJ, cervical spine, and shoulder girdle, thereby forming a functional unit that operates through coordinated muscular and neural mechanisms.

The stomatognathic system functions as part of this interconnected network, in which cervical posture, scapular alignment, and masticatory muscle activity are interdependent through shared muscular chains and neuromotor control mechanisms [3]. Disruption in one component, such as trauma affecting the scapulohumeral complex, may propagate along this kinetic chain, leading to compensatory cervical adaptations that influence masticatory muscle tone, TMJ loading, and mandibular biomechanics [4].

In this context, the relationship between scapulohumeral trauma and TMJ dysfunction has received increasing attention. Studies report a frequent coexistence of TMD and scapulohumeral dysfunction, particularly in cases involving trauma-related scapular dyskinesis [5]. This condition may contribute to shoulder impingement and reflect altered scapulohumeral function, thereby supporting the concept of a functional biomechanical link between these anatomical regions [6,7,8].

Scapulohumeral fractures may disrupt structural integrity and induce muscular and functional adaptations that affect posture and prolong recovery. Emerging evidence suggests that these changes may also influence TMJ function through shared biomechanical pathways, although a direct causal relationship has not been conclusively established. Therefore, the assessment of scapular dyskinesis may provide clinically relevant insight for therapeutic strategies in patients presenting with both shoulder injury and TMD [9,10].

Traumatic scapular dyskinesis is frequently associated with rotator cuff tears, proximal humerus fractures, Hill–Sachs lesions, and Bankart lesions. It may also coexist with internal or external impingement, rotator cuff tendinopathy, subacromial bursitis, biceps tendinopathy, acromioclavicular joint arthropathy, and scapulothoracic bursitis, and more rarely to scapular tumors. Secondary instability events have been reported more frequently following primary trauma, accounting for approximately 54.5% of subluxations and 33.3% of dislocations [11].

Current approaches to the diagnosis and management of scapular dyskinesis continue to evolve in parallel with advances in understanding the biomechanical and clinical role of the scapula in shoulder function. Increasing evidence highlights the importance of addressing shoulder dysfunction within rehabilitation strategies, particularly in patients presenting with pain, postural alterations, or trauma-related impairment [12,13].

In cases of scapulohumeral injury, a comprehensive evaluation should include assessment of the brachial plexus, axillary nerve, and distal vascular circulation, with particular attention to deltoid muscle activation. Vascular integrity is typically assessed using Doppler ultrasonography and computed tomographic angiography (CTA), whereas nerve involvement may be evaluated by electromyography, particularly in proximal humerus fractures [14,15,16]. Imaging techniques such as radiography, magnetic resonance imaging (MRI), computed tomography (CT), and ultrasound are essential for identifying traumatic lesions, evaluating associated conditions, and assessing soft tissue involvement.

Scapular dyskinesis represents a relevant impairment of shoulder function and should be addressed within a comprehensive therapeutic framework.

Rehabilitation strategies may improve scapular muscle coordination, optimized positioning, and support symptom control; however, these interventions should be integrated into multimodal and interdisciplinary rehabilitation approaches [17,18,19].

Posture reflects the functional integration of the skeletal and muscular systems and may be affected by pain, muscular imbalance, abnormal tension, and kinetic chain dysfunction. Non-functional posture is characterized by altered alignment across the three spatial planes and may manifest clinically as headaches, cervical pain, back pain, or limb discomfort. Contributing factors include inflammatory processes, congenital or acquired deformities, and traumatic injuries.

Given this complexity, the multimodal interdisciplinary rehabilitation plays an essential role and requires individualized treatment strategies tailored to each patient.

Therapeutic management may include physiotherapy, kinesiotherapy, surgical intervention when indicated, and comprehensive oral rehabilitation, with particular emphasis on prosthetic, periodontal, and orthodontic considerations [20].

The literature suggests that shoulder-girdle dysfunction, postural abnormalities, and TMD symptoms may be interconnected through shared biomechanical and neuromuscular pathways. However, current evidence does not support a direct causal relationship between scapulohumeral injury and TMJ dysfunction (Du et al., 2025 [21]; Botros et al., 2022 [22]; Manfredini et al., 2017 [23]; Costa et al., 2017 [24]).

Mandibular rehabilitation, including the management of the TMJ and masticatory muscles, plays an essential role in restoring cranio-mandibular function. Depending on the complexity of each case, treatment may include splint therapy, prosthetic rehabilitation, implant therapy, and adjunctive physical interventions aimed at muscle relaxation and tissue recovery [25,26,27].

However, the role of occlusion in TMD remains controversial. Systematic reviews have shown no clinically meaningful association between dental occlusion and TMD, thereby challenging traditional gnathological concepts (Manfredini et al., 2017 [23]). Furthermore, current evidence regarding the effectiveness of occlusal interventions remains limited and of low certainty [28]. Therefore, the clinical changes observed following oral rehabilitation cannot be attributed exclusively to occlusal correction, as neuromuscular adaptation, postural dynamics, and multimodal therapeutic interventions are also likely to contribute.

Recent studies suggest that comprehensive rehabilitation may contribute to enhanced muscular function, restoration of postural balance, and reduction in TMD-related dysfunction, thereby improving quality of life. However, results should be interpreted within a multifactorial and non-causal framework, particularly in observational study designs, in which multiple interacting variables may influence clinical outcomes [29,30,31,32,33,34].

The primary aim of this study was to assess whether patients with TMD and associated scapular dysfunction exhibit clinically relevant postural and functional alterations. The secondary aims were to characterize the pattern of scapular dyskinesis in patients with TMJ dysfunction, evaluate the relationship between TMJ symptoms and postural changes, and explore the implications of these findings for interdisciplinary rehabilitation strategies.

## 2. Materials and Methods

The study was designed as a retrospective observational study with prospective follow-up evaluations. Initial clinical data were obtained from the medical records of patients hospitalized with scapulohumeral fractures and subsequently managed through an interdisciplinary approach. Follow-up assessments were then conducted prospectively. Written informed consent was obtained from all patients before the prospective follow-up phase after a detailed explanation of the study aims, procedures, risks, and benefit.

The reporting of the study was conducted in accordance with the STROBE (Strengthening the Reporting of Observational Studies in Epidemiology) guidelines, which provide a standardized framework for the transparent and complete reporting of observational research. The manuscript was accordingly revised to ensure clarity in participant selection, variable definitions, follow-up procedures, statistical analysis, and study limitations.

The design was non-randomized and uncontrolled; therefore, the findings should be interpreted as associative rather than causal.

This study is an observational study that examines the impact of scapulohumeral fractures on the function of the temporomandibular joint (TMJ) and the associated postural changes. The study was conducted by analysing medical records of patients hospitalized with fractures and subsequently treated comprehensively for such injuries, with the aim of identifying correlations between postural dysfunctions and TMJ disorders. Forty-five participants were selected from a sample of patients treated between the years 2022–2025 for scapulohumeral fractures and other associated injuries. The study was conducted at the Department of Oral and Maxillofacial Surgery Clinic, and Orthopedics Department of Sf. Spiridon Hospital, and also, the Department of Prosthodontics, Faculty of Dental Medicine, Grigore T. Popa University of Medicine and Pharmacy, Iasi, Romania. The sample consisted of patients who already presented with symptoms of temporomandibular dysfunction and postural alterations. at the time of enrolment. Therefore, the study did not assess the incidence of TMD or postural alterations after fracture in the general population, but rather the evolution of these findings in a selected symptomatic cohort.

**Ethical Considerations**

The Ethics Committee of Grigore T. Popa University of Medicine and Pharmacy Iasi approved the protocol (Approval No. 176/17.04.2022). Written informed consent was obtained from all participants after detailed explanation of study aims, procedures, risks, and benefits. All participants were enrolled voluntarily, confidentiality was maintained in accordance with the principles of the Declaration of Helsinki, and no form of therapeutic coercion was applied.

### 2.1. Study Design and Patient Selection

The selection process followed the criteria below.

Inclusion criteria for the study were as follows: patients aged 18 to 65 years with scapulohumeral fractures confirmed by imaging investigations (X-ray, MRI, or CT) and with fractures older than six months. Participants were also required to exhibit temporomandibular symptoms and postural changes associated with the fracture. Because only patients with both TMJ symptoms and postural alterations were included, the sample was selective and cannot be used to estimate the prevalence of these findings among all patients with fractures.

Exclusion criteria included patients with major neurological conditions that could influence posture, a history of recent surgical interventions involving the TMJ or shoulder, rheumatologic diseases or other systemic conditions affecting the joints, a history of severe craniofacial trauma, and individuals with psychiatric disorders.

### 2.2. Clinical Timeline

The evaluations of patients already diagnosed and treated for scapulohumeral fracture included:

Clinical investigations: Examination of musculoskeletal function at the TMJ level, evaluation of posture.

The clinical timeline was defined as follows: baseline referred to the first interdisciplinary evaluation after orthopedic stabilization of the fracture and before initiation of oral rehabilitation; Eval. 1 (one month after) referred to the first follow-up after initiation of oral rehabilitation; Eval. 2 (three months after) referred to the mid-term re-evaluation; and Eval. 3 (six months after) referred to the final follow-up at the end of the observation period. TMD symptoms were documented as having developed or worsened after the scapulohumeral fracture, and the duration of symptoms at baseline was recorded for each patient.

### 2.3. TMJ Dysfunction Classification

Temporomandibular dysfunction was assessed clinically through a structured examination informed by the Diagnostic Criteria for Temporomandibular Disorders (DC/TMD) framework [34], including evaluation of joint sounds, mandibular range of motion, pain on palpation, and functional limitation. However, the categories ‘no dysfunction,’ ‘mild dysfunction,’ and ‘moderate dysfunction’ used in the present analysis were study-specific severity groupings intended for longitudinal description and do not correspond to formal DC/TMD diagnostic categories.

### 2.4. Paraclinical Postural Assessments

Scapulohumeral position and its relationship to overall posture were assessed using the Free Med™ baropodometric platform, which analyzes the static position of the scapula and shoulder girdle in relation to the body axis by means of anatomical landmarks and postural analysis software. The system can identify deviations such as scapular protraction, inferior or superior rotation, and anterior tilting.

When used in combination with FreeStep™ software, the Free Med™ baropodometric platform from Sensor medica, Rome, Italy, allows postural and biomechanical assessment (Figure S1). The system enables assessment of plantar pressure distribution, identification of weight-centre displacement, detection of high-pressure areas, and evaluation of asymmetries between the lower limbs. It may also assist in identifying scapular protraction, rotational alterations, and anterior scapular inclination, thereby providing information on compensatory postural adaptations and postural tone imbalances. Scapular analysis may reveal winging, asymmetry between the scapulae, and abnormal scapular rotation patterns. In addition, deviations in overall alignment, including misalignment of the spinous processes and pubic symphysis relative to the vertical body axis, may be observed. Combined findings such as muscular asymmetry, differences in muscle length affecting posture and movement, excessive plantar pressure on one side, and contralateral scapular displacement may suggest functional scoliosis with pelvic compensation.

Based on the global alignment patterns identified by the Free Med™/FreeStep™ system standard version from Medica sensor) and the clinician’s assessment of the magnitude and direction of observable postural deviation, postural changes were classified as slight forward lean, moderate forward lean, severe forward lean, or lateral lean. These categories were used as descriptive groupings for the purposes of this study and do not represent a formally validated severity scale. Rather than adopting a previously established literature-based grading system, the study used operational postural categories defined for descriptive analysis (Figure S2).

### 2.5. Functional Analyses

Functional analyses included electromyographic assessment of muscle activity and mobility testing of the temporomandibular joint (Figure S3a,b). Electromyographic evaluation was used to assess bilateral masseter and anterior temporalis muscle activity. Variables recorded included muscle activation pattern, contraction symmetry, and resting tonicity.

**Mandibular Mobility Measurements**

Mandibular mobility was assessed by measuring maximum voluntary mouth opening and lateral excursions. All examinations were performed by trained clinicians following standardized procedures.

### 2.6. Therapeutic Interventions

Therapeutic interventions were individualized and could include occlusal splint therapy, pharmacological treatment with muscle relaxants and non-steroidal anti-inflammatory drugs when indicated, physiotherapy and kinesiotherapy, and supportive periodontal rehabilitation where needed. Physiotherapy generally consisted of manual therapy, therapeutic exercises, and proprioceptive training performed two to three times per week for 8 to 12 weeks. Occlusal splints were worn nightly for at least three months when prescribed. The treatment protocol varied according to the severity of TMJ dysfunction, fracture type, postural deviation pattern, and associated clinical conditions.

### 2.7. Statistical Analysis

The statistical analysis was adapted to the categorical and ordinal nature of the data. Descriptive statistics were used to summarize the demographic and clinical characteristics of the sample. Associations between categorical variables were assessed with the Chi-square test or Fisher’s exact test, as appropriate when expected cell counts were small. Changes in paired categorical postural data across evaluation points were analyzed using the McNemar–Bowker or Stuart–Maxwell test. Repeated ordinal changes in TMD severity were assessed using the Friedman test, and pairwise post hoc comparisons were performed with the Wilcoxon signed-rank test. Between-group comparisons across treatment combinations were evaluated using the Kruskal–Wallis test. Statistical significance was set at *p* < 0.05. All analyses were performed using SPSS version 27, USA, New York.

## 3. Results

### 3.1. Demographic and Clinical Characteristics

The study included 45 patients with a mean age of 37.2 ± 7.02 years. Most participants were male (71.11%), while female participants accounted for 28.89% of the sample. The majority of patients were from urban areas (86.67%), whereas 13.33% were from rural areas. In terms of socioeconomic status, most patients were classified as having a high level (40.00%), followed by a medium level (37.78%) and a low level (22.2%).

The injuries analyzed included scapular fractures (31.11%), clavicle fractures and proximal humerus fractures (26.67% each), whereas acromioclavicular dislocation was the least frequent condition (15.56%). Overall, the distribution of injury types was relatively balanced, with scapular fractures being the most frequent (Table S1).

The cohort was predominantly male and urban, with scapular fractures representing the most frequent injury type.

### 3.2. Baseline Postural Classification

Statistical analysis of Table 1 indicates that, before treatment, the most common postural change in patients with various diagnosed dysfunctions was moderate forward lean (53.3%) (*n* = 24), followed by slight forward lean (26.7%) (*n* = 12), while severe and lateral leaning were less frequent (8.9% (*n* = 4) and 11.1% (*n* = 5), with variations between types of fractures and dislocations, thus highlighting a general tendency for anterior postural compensation (Table 1). No patient presented with normal posture at baseline.

Before treatment, moderate forward lean was the most common postural deviation (53.33%), followed by slight forward lean (26.67%), slight lateral lean (11.11%), and severe forward lean (8.89%). No patient presented with normal posture at baseline.

The distribution of postural deviations did not differ significantly by fracture type (Fisher’s exact test, *p* = 0.09), indicating that baseline postural impairment was present across all injury categories.

### 3.3. Postural Change After Treatment

After interdisciplinary rehabilitation overall postural alignment improved (descriptive data are shown in Supplementary Table S2). The proportion of patients with normal posture increased from 0.0% to 22.2% (*n* = 10), while moderate forward (fwd)- lean decreased from 53.3% (*n* = 24) to 15.6% (*n* = 7). Severe forward (fwd)- lean also decreased slightly, from 8.9% (*n* = 4) to 4.4% (*n* = 2), and slight lateral (lat)- lean decreased from 11.1% (*n* = 5) to 2.2% (*n* = 1) (Table 2).

The post-treatment distribution shifted toward more favorable postural categories, with the most notable changes being *the reduction in moderate forward (fwd) lean* and the emergence of normal posture in a substantial subset of patients. This change in paired categorical data was statistically significant, indicating a clear shift in postural distribution over time. However, because the study was observational and non-randomized, these changes cannot be attributed solely to the treatment interventions, as natural recovery, concurrent orthopedic rehabilitation, and regression to the mean may also have contributed to the observed improvement.

### 3.4. Post-Treatment Postural Classification by Fracture Type

Post-treatment postural distribution was broadly comparable across injury types (Fisher’s exact test, *p* = 0.178); detailed subgroup data are presented in Supplementary Table S3.

Patients with scapular fracture showed the highest proportion of recovery to normal posture (35.7%, *n* = 5/14), whereas no patients with proximal humerus fractures were classified as having normal posture at follow-up.

Post-treatment postural distribution was similar across injury types, with no significant between-group differences observed. The highest rate of recovery to normal posture was observed in the scapular fracture subgroup, although this finding should be considered exploratory.

### 3.5. Evolution of TMD Severity Across Evaluations

TMJ dysfunction improved progressively across the four evaluation points (Table 3). At baseline, 68.9% (*n* = 31) of patients had moderate dysfunction and 31.1% (*n* = 14) had mild dysfunction. At Eval 1 (one month), most patients shifted to the mild dysfunction category (80.0%, *n* = 36), while moderate dysfunction decreased to 20.0% (*n* = 9). At Eval 2 (three months), 31.1% (*n* = 14) of patients had no TMJ dysfunction, 62.2% (*n* = 28) had mild dysfunction, and only 6.7% (*n* = 3) had moderate dysfunction. At Eval 3 (six months), 71.1% (*n* = 32) of patients had no dysfunction, 28.9% (*n* = 13) had mild dysfunction, and moderate dysfunction was no longer observed.

The Friedman test showed that this change was statistically significant (χ^2^ = 72.35, *p* < 0.01).

The main longitudinal finding of the study was the progressive reduction in moderate dysfunction together with an increase in the number of patients without dysfunction (Figure S4).

### 3.6. Pairwise Changes in Temporomandibular Dysfunction

Pairwise comparisons across consecutive evaluations were analyzed using the Wilcoxon signed-rank test (Table 4). All comparisons showed a statistically significant trend toward improvement (*p* < 0.01), indicating that the change was progressive rather than limited to a single time point.

Repeated TMD assessments showed a consistent trend toward improvement at each follow-up point. Overall, recovery appeared to be gradual and progressive throughout the evaluation period.

### 3.7. Postural Changes by Treatment Combination

Descriptive data on postural change according to treatment combination are presented in Supplementary Table S3. The Kruskal–Wallis test showed no statistically significant difference in postural outcomes across treatment groups (*p* = 0.103). Because treatment allocation was not randomized and groups were small and heterogeneous, these comparisons should be regarded as exploratory and should not be interpreted as evidence of differences in treatment effect.

The descriptive data suggest that more complex treatment pathways were associated with greater postural improvement. However, patients receiving more complex treatment were likely to have differed at baseline with respect to dental status, occlusal status, symptom severity, and general rehabilitation needs. Accordingly, these findings should not be interpreted as evidence of treatment superiority, because the analyses were exploratory and potentially confounded by small cell counts and baseline differences in severity.

The analysis was limited to descriptive statistics; Kruskal–Wallis test exploratory (*p* = 0.103 for post-recovery posture).

### 3.8. Temporomandibular Dysfunction Changes by Treatment Combination

Descriptive analysis of changes in TMJ dysfunction according to treatment combination is presented in Supplementary Table S4. Across all groups, TMJ severity declined over the follow-up period, with the overall mean decreasing from 1.69 at baseline to 0.29 at Eval. 3. However, the study design does not permit this change to be attributed to any single treatment strategy.

A similar pattern was observed for TMJ dysfunction (Table S4). TMJ severity decreased over the follow-up period in nearly all treatment combinations, with the lowest final scores seen in the multimodal treatment groups. The mean TMJ score declined steadily across evaluations, from 1.69 at baseline to 0.29 at Eval. 3, indicating progressive improvement over time.

TMJ recovery followed a consistent downward trajectory in severity across the cohort. This supports the broader conclusion that the cohort improved over the follow-up period, although the study design does not allow attribution of the change to any single treatment strategy.

The analysis was limited to descriptive statistics; Kruskal–Wallis test exploratory.

### 3.9. Recovery Outcomes and Associated Pathology

Recovery outcomes stratified by fracture type and systemic pathology are presented in Supplementary Table S5. Because of the limited cell counts, these findings should be interpreted as exploratory rather than inferential.

Recovery outcomes were generally more favorable in patients without systemic pathology (Table S5). Patients with systemic pathology were more likely to fall into the less favorable outcome categories, although the number of such cases was small.

The data suggest a possible negative influence of systemic pathology on recovery, but the evidence is limited by the small subgroup sizes and small cell counts. This finding should be considered exploratory and should be confirmed in larger studies.

Statistical approach: Fisher’s exact test (exploratory, sparse cells).

In summary, the study showed progressive changes in both posture and TMJ dysfunction over the follow-up period. Postural improvement was most notable in the reduction in moderate forward lean and the emergence of normal posture, while TMJ dysfunction shifted steadily from moderate to mild or no dysfunction. Descriptive analyses across treatment groups suggested greater benefit in some multimodal treatment pathways; however, these findings remain exploratory because the groups were small, heterogeneous, and not randomized. The observed changes therefore reflect longitudinal associations within a selected symptomatic cohort rather than evidence of a causal treatment effect.

## 4. Discussion

This study suggests that scapulohumeral fractures may be associated with alterations in TMJ function and postural balance, while interdisciplinary rehabilitation contributes to functional recovery. These findings support the concept of regional interdependence, whereby dysfunction in one anatomical region may influence the function of related biomechanical systems.

A progressive enhancement in both postural alignment and TMJ function was observed over the follow-up period in patients recovering from scapulohumeral injury. Although clinically relevant, these findings should be interpreted as associations observed within a selected symptomatic cohort rather than as evidence of a direct causal relationship (List et al., 2017 [35]; Costa et al., 2015 [36]; do Nascimento et al., 2020 [37]).

Contemporary literature supports a multifactorial model of TMD, in which biological, biomechanical, neuromuscular, and psychosocial factors interact, while traditional occlusion-centered theories have progressively lost explanatory relevance (Gagey et al., 2019 [38]; de Oliveira-Souza et al., 2020 [39]).

At baseline, the cohort was predominantly male, largely urban, and clinically heterogeneous, with scapular fractures representing the most frequent injury type. This distribution is in line with previous research indicating that shoulder-girdle trauma may impair scapular control, alter cervical posture, and trigger compensatory neuromuscular adaptations. Kibler et al. (2012 [40]) highlighted scapular dyskinesis as a clinically relevant factor in shoulder injury, and subsequent work further emphasized its role in shoulder instability and movement control (Kibler and Sciascia, 2016 [41]). In addition, Haik et al. (2014 [41,42]) demonstrated that scapular orientation can be assessed reliably, underscoring its importance in rehabilitation research.

At baseline, moderate forward lean was the most frequent postural alteration, followed by less severe deviations, whereas marked imbalance was less common. This pattern is consistent with literature linking forward head posture and scapular protraction to musculoskeletal dysfunction. Carini et al. (2017 [20]) described posture as the result of integrated anatomical and physiological regulation, while other studies have associated scapular dyskinesis with altered cervical mechanics and chronic neck symptoms (Gagey et al., 2019 [38]). These observations support the hypothesis that shoulder-girdle trauma may induce compensatory postural adaptations extending beyond the primary site of injury [43,44,45,46].

The absence of statistically significant differences between fracture subgroups suggests that postural alterations were not specific to a given injury type, but instead reflected a more generalized adaptive response to upper-limb dysfunction. These findings should therefore be interpreted as descriptive and not as evidence of a direct mechanistic relationship between fracture type and TMJ dysfunction [47,48,49].

Following treatment, postural alignment improved, as reflected by an increased proportion of patients with normal posture and a reduction in forward lean. This pattern is in line with studies suggesting that postural training and cervical stabilization may support functional improvement and reduce the burden of TMD-related symptoms [50,51].

However, these changes should not be attributed exclusively to oral rehabilitation. The observational design, lack of randomization, and absence of a control group limit causal interpretation, and spontaneous recovery may have also contributed to the observed outcomes. This is particularly relevant given that current evidence does not support a purely occlusion-driven model of TMDs. Manfredini et al. (2017 [23]) reported no clinically meaningful association between TMDs and dental occlusion, while Singh et al. (2017 [52]) found insufficient evidence to support the effectiveness of occlusal interventions.

Post-treatment posture remained broadly similar across injury subgroups, although patients with scapular fractures showed a higher proportion of recovery to normal posture. Because this difference was not statistically significant and subgroup sizes were limited, these findings should be interpreted cautiously. Overall, postural adaptation appears to reflect general rehabilitation processes rather than a diagnosis-specific effect.

TMJ dysfunction showed a consistent reduction in severity across follow-up evaluations, with most patients reaching a symptom-free status by the final assessment. This trajectory is clinically meaningful and is consistent with the literature supporting conservative and multimodal approaches in TMD management. de Leeuw and Klasser (2015 [53]) emphasized the importance of comprehensive orofacial pain management, while Schiffman et al. (2014 [54]) established the DC/TMD framework for standardized diagnosis. Valesan et al. (2021 [55]) further highlighted the high prevalence and clinical burden of TMD.

Importantly, these findings should not be interpreted as evidence for an occlusal etiology of temporomandibular disorders. Within the current multifactorial framework of TMD, occlusion is regarded as one of several contributing factors rather than a primary causal determinant. The contemporary literature consistently supports this view, emphasizing the limited and non-deterministic role of occlusal factors in TMD pathogenesis. In particular, Manfredini et al. (2017 [23]) reported no clinically meaningful association between occlusion and TMD, while Singh et al. (2017 [52]) found only the low-certainty evidence regarding the effectiveness of occlusal interventions.

Accordingly, the changes observed in the present study are more appropriately interpreted as reflecting adaptive neuromuscular responses and the cumulative influence of multidisciplinary therapeutic strategies, rather than as effects attributable solely to occlusal correction.

Furthermore, the progressive reduction in symptom severity across follow-up evaluations is consistent with the pattern typically reported in studies of conservative treatment, in which clinical improvement emerges gradually over time. This trajectory supports the role of physiotherapy, postural retraining, and self-management as relevant components of TMD management. Earlier work by Wright et al. (2000 [44]) showed that integrating postural interventions with self-management strategies was associated with improved clinical outcomes, thereby reinforcing the value of a comprehensive multimodal treatment approach.

Multimodal treatment approaches appeared to offer greater descriptive benefit, particularly when splint therapy, pharmacological management, physiotherapy, and supportive periodontal care were combined. This pattern may reflect a synergistic therapeutic effect, as multiple contributing factors are addressed simultaneously. Zhang et al. (2025 [56]) reported that occlusal splints may be beneficial in selected cases, while recent reviews continue to support conservative, reversible interventions as first-line approaches.

However, these subgroup findings should be interpreted with caution. Because the treatment groups were small, heterogeneous, and non-randomized, the observed differences should not be interpreted as evidence of treatment superiority, as they may instead reflect baseline severity or underline clinical variability.

Patients without systemic pathology appeared to have more favorable recovery trajectories, whereas those with systemic conditions showed less pronounced functional improvement. Although biologically plausible, these findings remain exploratory and require validation in larger cohorts [57].

One possible explanatory model is that shoulder-girdle injury alters scapular mechanics and cervical posture, leading to secondary neuromuscular adaptations that may influence masticatory function and TMJ dynamics. The present findings support a possible association between scapulohumeral injury and TMJ dysfunction, consistent with the concept of regional interdependence. However, the present study does not establish causality and only shows parallel changes in posture and TMJ status over time.

Overall, the results suggest that the observed changes reflect a broader multidisciplinary rehabilitation process rather than a single therapeutic mechanism.

Occlusion should therefore be regarded as one of several contributing factors rather than as the primary etiological determinant [57,58,59,60,61,62].

Multimodal interdisciplinary rehabilitation may contribute to functional recovery in patients with TMD and scapulohumeral injury. These findings further support the view that TMJ dysfunction should be understood within an integrated neuromuscular system rather than as an isolated condition (Ohrbach and Dworkin, 2016 [63]).

## 5. Conclusions

- Interdisciplinary rehabilitation was associated with significant improvements in both postural alignment (McNemar–Bowker test, *p* < 0.01) and TMD severity (Friedman test, χ^2^ = 72.35, *p* < 0.01) in patients with scapulohumeral fractures and concomitant temporomandibular dysfunction. Clinical implication: Multimodal rehabilitation protocols simultaneously addressing to musculoskeletal and orofacial dysfunction should be integrated into the standard management of patients presenting with this clinical type association.
- A progressive reduction in TMD severity was observed across successive evaluation with 71.11% of patients (*n* = 32) showing no dysfunction at the final evaluation. Clinical implication: Sustained interdisciplinary intervention yields measurable, clinically benefits, supporting early orofacial screening and co-management within orthopedic rehabilitation programs.
- Pairwise comparisons using the Wilcoxon signed-rank test showed statistically significant changes at each consecutive stage(all *p*-value < 0.01), indicating a consistent trajectory of functional recovery. Clinical implication: The consistency of improvement across time points reinforces the therapeutic value of structured, sequential follow-up protocols in this patient group.
- The parallel improvement in postural alignment and attenuation of TMD symptoms suggests a functional interaction within the cranio-cervico-mandibular system, supporting the hypothesis of shared biomechanical and neuromuscular pathways linking the shoulder girdle, cervical spine, and TMJ. Clinical implication: Clinicians managing scapulohumeral injuries should routinely screen for temporomandibular dysfunction, as postural and orofacial dysfunctions may mutually reinforce one another and require concurrent therapeutic attention.
- However, given the multifactorial nature of TMD, the selectively recruited cohort, and the non-randomized design, these findings should be interpreted as associative rather than causal.
- No statistically significant differences were identified between groups for postural outcomes (Kruskal–Wallis, *p* = 0.103), although descriptive variations in TMD evolution were observed at later evaluation points. Clinical implication: These exploratory findings highlight the need for future randomized controlled trials to isolate the specific contributions of individual rehabilitation components.
- These between-group comparisons should be regarded as exploratory, as treatment allocation was not randomized and potential confounding factors cannot be excluded. Clinical implication: The presence of comorbidities should be considered when planning rehabilitation timelines and setting patient expectations for functional recovery.
- A descriptive trend suggested that systemic pathologies may negatively influence rehabilitation trajectories, although this observation did not reach statistical significance. Multimodal interdisciplinary rehabilitation may contribute to functional recovery in patients with TMD and scapulohumeral injury. Clinical implication: The presence of systemic pathologies should be factored into individualized rehabilitation planning and prognosis communication with patient.

As findings derive from a non-randomized design with a selectively recruited cohort, results should be interpreted as associative rather than causal. Clinical implication: Multimodal interdisciplinary rehabilitation represents a clinically promising approach for patients with concurrent TMD and scapulohumeral injury, warranting confirmation through prospective, controlled study designs with larger and more heterogeneous population.

Limitations

A major limitation of this study is the relatively small sample size (*n* = 45), which restricts statistical power, particularly for subgroup analyses according to fracture type, associated systemic pathology, and treatment combination. As a result, these comparisons were underpowered and should be interpreted with caution. Accordingly, all analyses comparing treatment combinations were considered exploratory and descriptive rather than confirmatory, and no causal or superiority inferences should be drawn from these findings.

The study population was restricted to patients who already presented with TMD symptoms and postural alterations. Therefore, the present study cannot determine whether scapulohumeral fractures cause TMD in the general population.

In addition, the non-randomized observational design limits causal inference and treatment heterogeneity with the lack of randomized allocation prevents isolation of specific treatment effects.

Psychosocial factors, such as stress, behavioral patterns, and adherence to therapy, were not systematically quantified and may have acted as confounding variables, as highlighted by Friesen et al. (2025) [64].

Furthermore, the FreeMed/FreeStep system does not directly measure scapular kinematics and scapular alignment was inferred from global postural analysis. Finally, the follow-up period was limited to short- and medium-term evaluations; therefore, long-term stability and relapse rates could not be assessed.

Future Directions

Future research should include large-scale randomized controlled trials with standardized interdisciplinary rehabilitation protocols in order to provide stronger evidence regarding causality. Extended longitudinal follow-up will also be necessary to assess the long-term stability of postural improvement and TMD-related outcomes. In addition, the use of advanced diagnostic tools, such as three-dimensional motion capture systems and biomechanical modeling, may offer deeper insights into the compensatory mechanisms involved in the cranio-cervico-mandibular system.

## Figures and Tables

**Table 1:** Type of postural change before treatment.

Diagnosis of Dysfunctions	TYPE of Postural Change Before Treatment				Fisher’s Test *p*
	Slight Fwd.-Lean	Moderate Fwd.-Lean	Severe Fwd.-Lean	Slight Lat.-Lean	
Clavicle Fracture	41.67%	25.00%		33.33%	0.09 (n.s.)
Scapular Fracture	21.43%	57.14%	14.29%	7.14%	
Proximal Humerus Fractures	16.67%	66.67%	16.67%		
Acromioclavicular Dislocations	28.56%	71.43%			
TOTAL (n = 45)	26 67%	53.33%	8.89%	11.11%	

**Table 2:** Postural distribution: baseline vs. post-treatment (*n* = 45).

**Postural Category**	**Baseline (%)**	**Post-Treatment (%)**	**Change (Pp)**
Normal Posture	0.00	22.22	+22.22
Slight Forward (fwd.) lean	26.67	55.56	+28.89
Moderate Forward (fwd.) lean	53.33	15.56	−37.78
Severe Forward (fwd.) lean	8.89	4.44	−4.45
Slight Lateral (lat.) lean	11.11	2.22	−8.89

Statistical test: McNemar–Bowker test, *p* < 0.01. The most important improvement was the reduction in moderate forward lean and the emergence of normal posture in a substantial proportion of patients.

**Table 3:** Evolution of TMD severity across evaluations (% of patients, *n* = 45).

**TMD Severity**	**Initial *n* (%)**	**Eval. 1 *n* (%)**	**Eval. 2 *n* (%)**	**Eval. 3 *n* (%)**
No TMJ dysfunction	**0 (0.0)**	**0 (0.0)**	**14 (31.1)**	**32 (71.1)**
Mild TMJ dysfunction	**14 (31.1)**	**36 (80.0)**	**28 (62.2)**	**13 (28.9)**
Moderate TMJ dysfunction (Joint Click, Dislocation or Subluxation, Pain)	**31 (68.9)**	**9 (20.0)**	**3 (6.7)**	**0 (0.0)**

**Table 4:** Pairwise change in TMD severity across evaluations (Eval.).

**Comparison**	**Statistical Test**	**Result**	***p*** **-Value**
Initial vs. Eval. 1	Wilcoxon Signed-Rank Test	Improvement	<0.01
Eval. 1 vs. Eval. 2	Wilcoxon Signed-Rank Test	Improvement	<0.01
Eval. 2 vs. Eval. 3	Wilcoxon Signed-Rank Test	Improvement	<0.01
Initial vs. Eval. 3	Wilcoxon Signed-Rank Test	Largest Improvement	<0.001

## Data Availability

Data supporting the reported results are available from the corresponding author upon reasonable request.

## Supplementary Materials

The following supporting information can be downloaded at: https://www.mdpi.com/article/10.3390/jcm15103597/s1. Figure S1. Examples of postural alterations identified through digital assessments in patients with fractures and associated postural alterations; Figure S2. Visual workflow of Free Med™ assessment; Figure S3. Mandibular movement trajectories during TMJ opening and closing, showing the right joint (blue) and left joint (red) under four different conditions or at four time points (panels 1–4). Electromyographic (EMG) recording of muscle activity during a functional task illustrating mandibular opening and closing movements or a controlled voluntary contraction; Figure S4. TMD severity evolution across evaluations (N = 45). Friedman χ^2^ = 72.35, *p* < 0.01; Table S1 Descriptive general data (N = 45); Table S2. Descriptive summary of postural changes by treatment combination; Table S3. Post-treatment postural classification by fracture type; Table S4. Descriptive summary of TMJ changes by treatment type combination. Table S5. Recovery outcomes by type of dysfunction and associated pathology.

## Abbreviations

*p*	*p*-value
TMJ	Temporomandibular joint
TMD	Temporomandibular disorders
TMDs	Temporomandibular disorders
SS	Stomatognathic system
EMG	Electromyography
MRI	Magnetic resonance imaging
SICK	Scapular Index, Cervical lordosis, Kyphosis scale
IBS	Irritable Bowel Syndrome
CSD	Cervical spine disease
FB	Fibromyalgia
MWU	Mann–Whitney U test
WSR	Wilcoxon signed-rank test
KW	Kruskal–Wallis test
FR	Friedman test
SIC	Scapular-humeral injuries
SD	Scapular dyskinesis
DC/TMD	Diagnostic Criteria for Temporomandibular Disorders
Fwd. (lean)	Forward lean (body tilted forward)
Lat. (lean)	Lateral lean (body shifted left or right)
