# Supplementary material for: The Impact of Complex Oral Rehabilitation on TMJ and Postural Alterations in Patients with Scapulohumeral Fractures
Source: J Clin Med. 2026 May 8;15(10):3597. doi: 10.3390/jcm15103597 (PMC13207656; doi:10.3390/jcm15103597)
Supplement: Supplementary file 1 [file jcm-15-03597-s001.zip › jcm-4205815-supplementary.pdf]

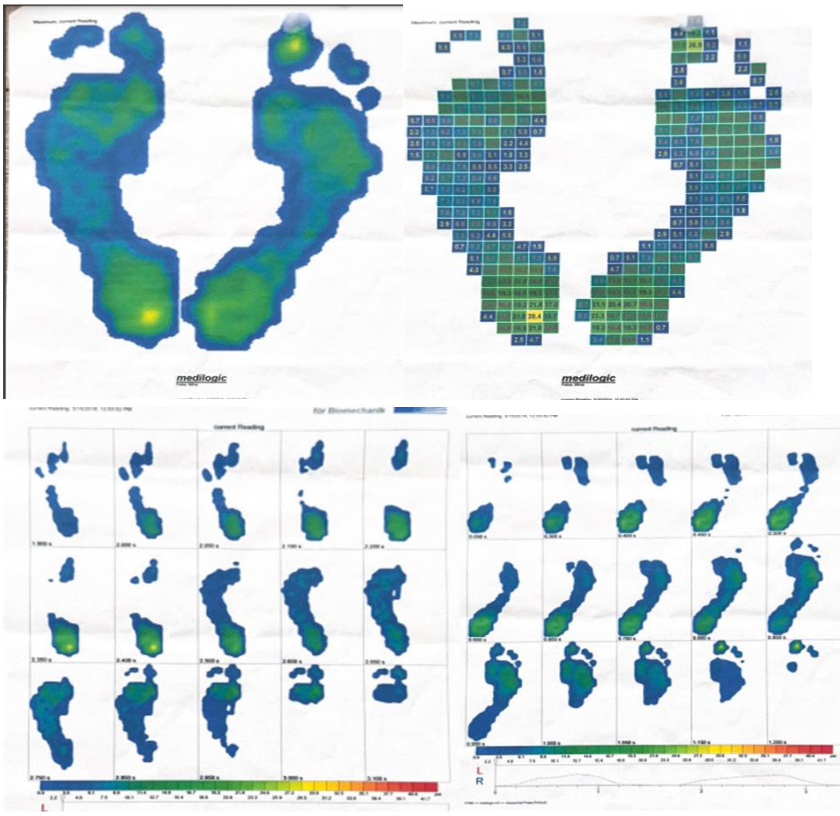

Figure S1. Examples of postural alterations identified through digital assessments in patients with fractures and associated postural alterations.

These analyses may support clinical decision-making by informing the selection of appropriate therapeutic strategies, including postural re-education and biomechanical corrective interventions such as strengthening exercises, mobility training, and neuromuscular retraining. The evaluation includes analysis of static plantar pressure distribution, identification of key anatomical landmarks, and detection of potential axial deviations in body alignment.

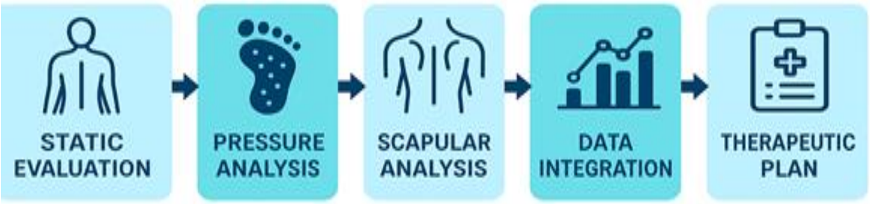

Figure S2. Visual workflow of Free Med™ assessment.

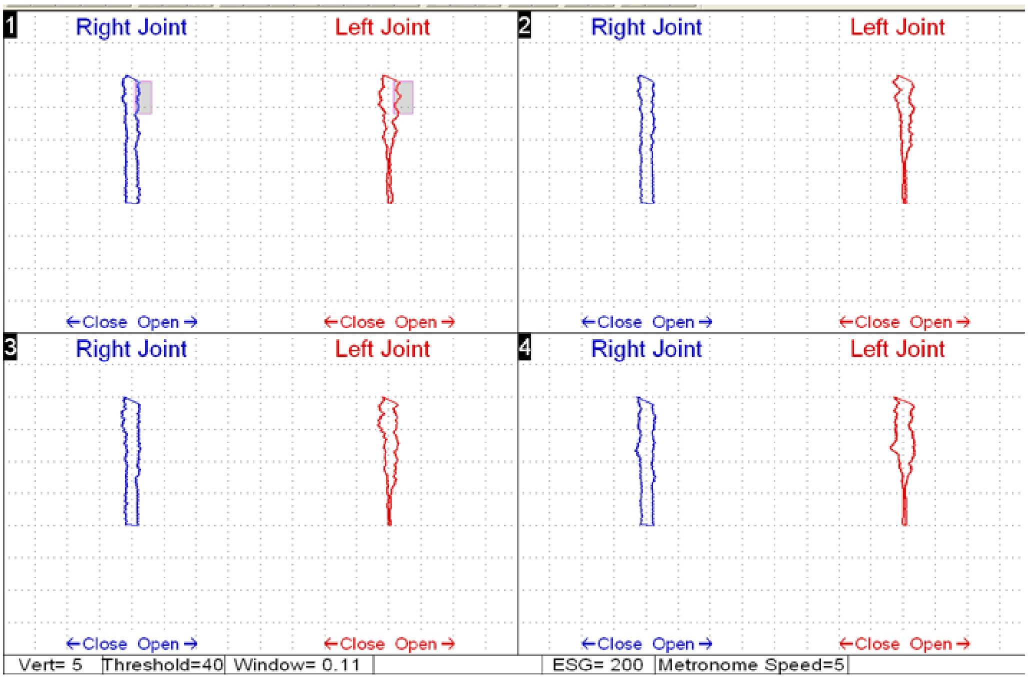

Figure S3a. Mandibular movement trajectories during TMJ opening and closing, showing the right joint (blue) and left joint (red) under four different conditions or at four time points (panels 1–4).

These graphical representations are used in clinical kinematic assessment to evaluate movement symmetry, coordination, and amplitude.

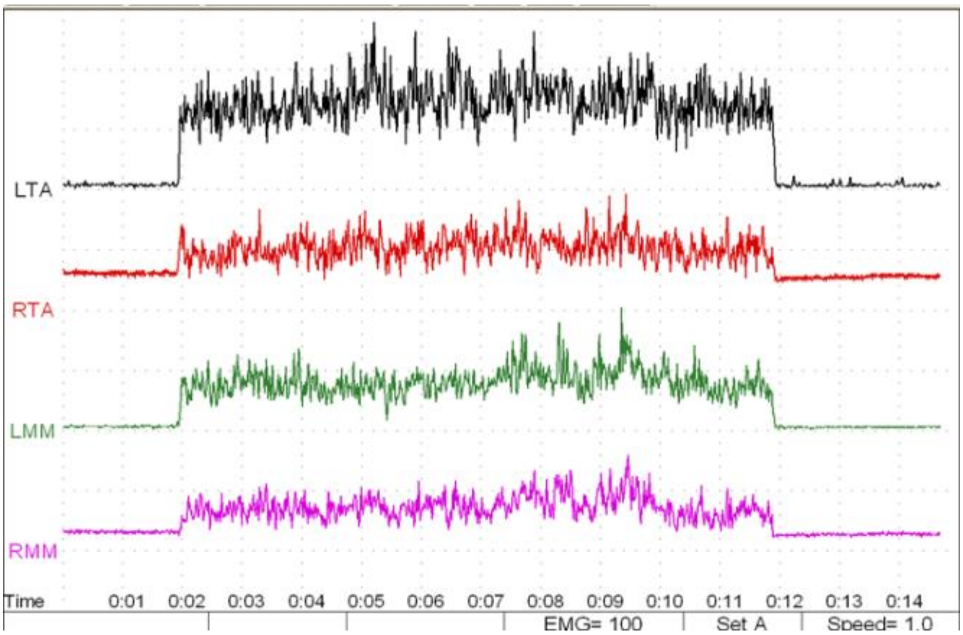

Figure S3b. Electromyographic (EMG) recording of muscle activity during a functional task illustrating mandibular opening and closing movements or a controlled voluntary contraction.

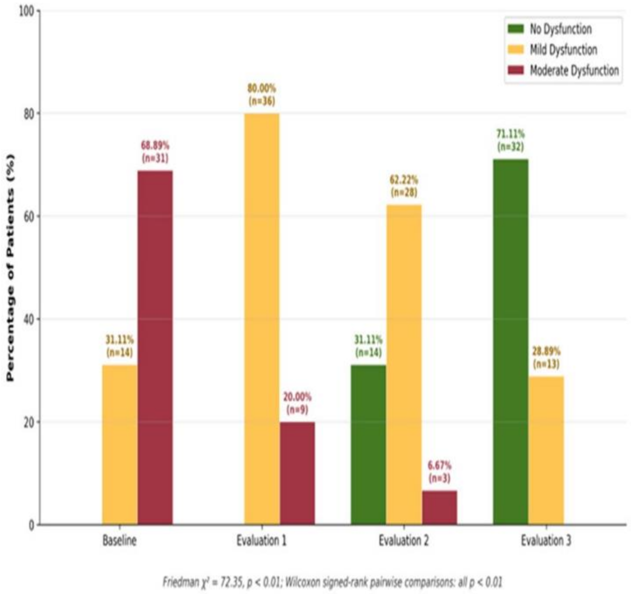

Figure S4. TMD severity evolution across evaluations (N = 45). Friedman $\chi^2 = 72.35$, $p < 0.01$.

Supplementary Tables S1–S5

Table S1 Descriptive general data (N=45).

Category /subgroups	N	%
**Age**	37.2± 7.02(Min Age 24 Years Old -Max.Age 56 Years Old)	
**Gender**		
**Female**	13	28.89
**Male**	32	71.11
**Residence**		
**Urban**	39	86.67
**Rural**	6	13.33
**Socioeconomic status**		
**High level**	18	40.00
**Medium level**	17	37.78
**Low level**	10	22,22
**Disfunction**		
**Clavicle fracture**	12	26.67
**Scapular fracture**	14	31.11
**Proximal humerus fracture**	12	26.67
**Acromioclavicular dislocation**	7	15.56

Table S2. Descriptive summary of postural changes by treatment combination.

Treatment combination	n	Mean before treatment	Mean after recovery	Direction of change
Splints + muscle relaxant medications	2	3.00	1.50	Improvement
Splints + medications + adjunct treatment	2	2.00	2.00	No clear change
Splints + medications + adjunctive treatment	10	2.20	1.67	Improvement
Splints + medications + odonto-periodontal treatment	7	2.14	1.00	Improvement
Splints + medications + concurrent and adjunctive treatment	4	1.50	0.75	Improvement
Splints + medications + concurrent treatment and odonto-periodontal treatment	4	2.00	1.75	Small improvement
Splints + medications + concurrent treatment, adjunctive treatment and odonto-periodontal treatment	8	2.13	0.88	Marked improvement
Splints + medications + adjunctive treatment, odonto-periodontal treatment and implant	1	1.00	0.00	Greatest improvement

.

Table S3 . Post-treatment postural classification by fracture type.

Injury Type	Normal Posture	Slight fwd Lean	Moderate fwd Lean	Severe fwd Lean	Slight lat. Lean	Fisher's Exact Test P
**Injury type**	**Normal posture**	**Slight fwd lean**	**Moderate fwd lean**	**Severe fwd lean**	**Slight lat.lean**	**Fisher's exact test p**
**Clavicle fracture (N=12)**	25.00%	33.33%	—	33.33%	8.33%	**0.178**
**Scapula fracture (N=14)**	35.71%	42.86%	14.29%	7.14%	—	
**Proximal humerus fracture (N=12)**	—	66.67%	25.00%	8.33%	—	
**Acromioclavicular dislocation (N=7)**	28.57%	57.14%	14.3%	—	—	
**Total (N=45)**	22.22%	48.89%	13.33%	13.33%	2.22%	

Table S4

Table S4. Descriptive summary of TMJ changes by treatment type combination.

Treatment combination	n	Mean Initial	Mean Eval. 1	Mean Eval. 2	Mean Eval. 3
Splints + muscle relaxant medication	2	2.00	1.00	0.00	0.00
Splints + medication + adjunct treatment	2	2.00	1.50	0.50	0.50
Splints + medication + combined treatment	15	2.00	1.27	0.87	0.20
Splints + medication + odonto-periodontal treatment	7	1.71	1.14	0.43	0.14
Splints + medication + combined and adjunct treatment	4	1.25	1.00	1.00	0.00
Splints + medication + combined treatment and odonto-periodontal treatment	4	1.50	1.25	0.75	0.00
Splints + medication + combined treatment, adjunctive treatment and odonto-periodontal treatment	8	1.50	1.25	1.25	1.00
Splints + medication + adjunctive treatment, odonto-periodontal treatment and implant	1	1.00	1.00	0.00	0.00
Total	45	1.69	1.20	0.76	0.29

Table S5. Recovery outcomes by type of dysfunction and associated pathology.

Recovery outcome	Pathology status	Clavicle fracture	Scapula fracture	Proximal humerus fracture	Acromioclavicular dislocation	Total
Very good	Without systemic pathologies	7 (36.8%)	2 (10.5%)	5 (26.3%)	5 (26.3%)	19
Very good	With systemic pathologies	0	0	1 (100%)	0	1
Good	Without systemic pathologies	3 (21.4%)	4 (28.6%)	5 (35.7%)	2 (14.3%)	14
Good	With systemic pathologies	2 (40.0%)	2 (40.0%)	1 (20.0%)	0	5
Unsatisfactory	Without systemic pathologies	0	1 (100%)	0	0	1
Unsatisfactory	With systemic pathologies	0	5 (100%)	0	0	5
